# Supplementary material for: Slow update of internal representations impedes synchronization in autism
Source: Nat Commun. 2021 Sep 14;12:5439. doi: 10.1038/s41467-021-25740-y (PMC8440645; doi:10.1038/s41467-021-25740-y)
Supplement: Supplementary file 1 — Supplementary Information [file 41467_2021_25740_MOESM1_ESM.pdf]

Slow update of internal representations impedes synchronization in autism – supplementary material

Vishne et al., 2021

Supplementary Note 1. Test re-test reliability of basic tapping measures

All conditions were repeated twice, and we compared the two repetitions. To form test re-test measurements of all blocks we averaged across conditions, after z-scoring each condition separately (using the mean and standard deviation of the neurotypical group). Both the standard deviation (SD) of asynchronies and the mean asynchrony were highly reliable both when considering each group separately and when pooling across groups (all correlations were highly significant, p<0.0001). For the SD the Spearman correlations were $\rho_{CON} = 0.8$, $\rho_{DYS} = 0.9$ and $\rho_{ASD} = 0.94$ and for the mean asynchrony they were $\rho_{CON} = 0.72$, $\rho_{DYS} = 0.85$ and $\rho_{ASD} = 0.92$ (Supplementary Figure 1). Pooling across groups the Spearman correlations were $\rho_{ALL} = 0.9$ for standard deviation and $\rho_{ALL} = 0.83$ for mean asynchrony.

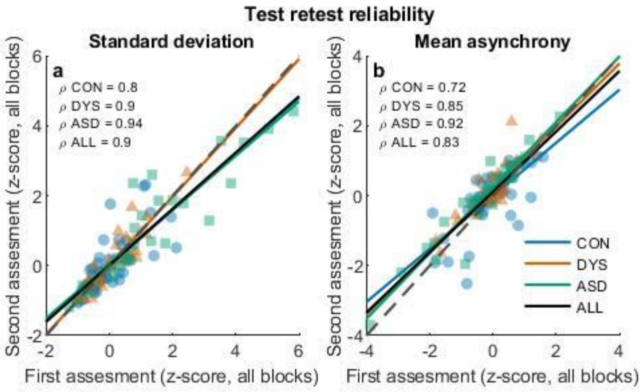

Supplementary Figure 1. Test-retest reliability of the basic tapping measures. (a) Standard deviation of the asynchronies is highly correlated between the two repetitions in all groups (CON – control (neurotypical), DYS – dyslexia, ASD – autism), with Spearman correlations $\rho_{CON} = 0.8$, $\rho_{DYS} = 0.9$ and $\rho_{ASD} = 0.94$. (b) Mean asynchrony is highly correlated between the two repetitions in all groups, with Spearman correlations $\rho_{CON} = 0.72$, $\rho_{DYS} = 0.85$ and $\rho_{ASD} = 0.92$. Each dot represents the first (x-axis) and second (y-axis) assessments of one participant. The regression line of each group is denoted as a line of the same color. The dashed line represents equal values for the two assessments (test=retest). N=109 subjects (N_{CON}=47, N_{DYS}=32, N_{ASD} =30). Source data are provided as a Source Data file.

Supplementary Figures 2-3. Basic tapping parameters in all conditions

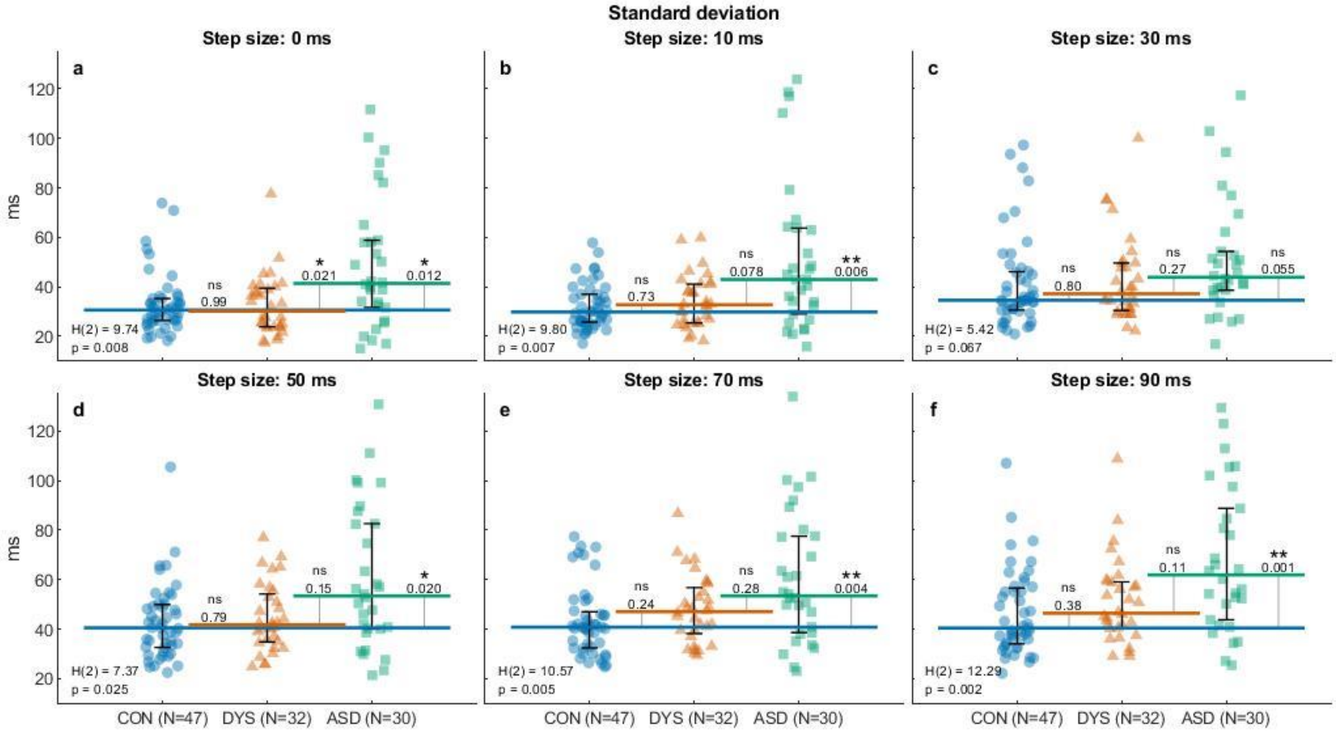

Supplementary Figure 2. Standard deviation (SD) is significantly larger in the ASD group compared with the two other groups in both experiments. (a) Experiment 1: no tempo change (0 step-size). (b-f) Experiment 2: tempo changes, in step-sizes from 10ms to 90ms. There is a significant group difference for all step-sizes (except 30ms where the difference is marginal), stemming from increased values in the ASD group. Each dot represents the standard deviation of one participant in a specific condition (average of the two repetitions). x-axis and color represent group membership (CON – control (neurotypical), DYS – dyslexia, ASD – autism; a small jitter was added to improve readability). The median of each group is denoted as a line of the same color; error bars around this median denote interquartile range. Kruskal-Wallis H-statistic and corresponding p-value are in the bottom-left corner; p-values of comparisons between groups are next to the line connecting the groups' medians. P-values in the panels were not corrected for multiple comparisons across conditions. A combined SD value (formed by z-scoring in each condition using the neurotypical mean and standard deviation and then averaging) also showed a significant group difference (Kruskal-Wallis test $H(2)=10.32$, $p<0.006$). Source data are provided as a Source Data file.

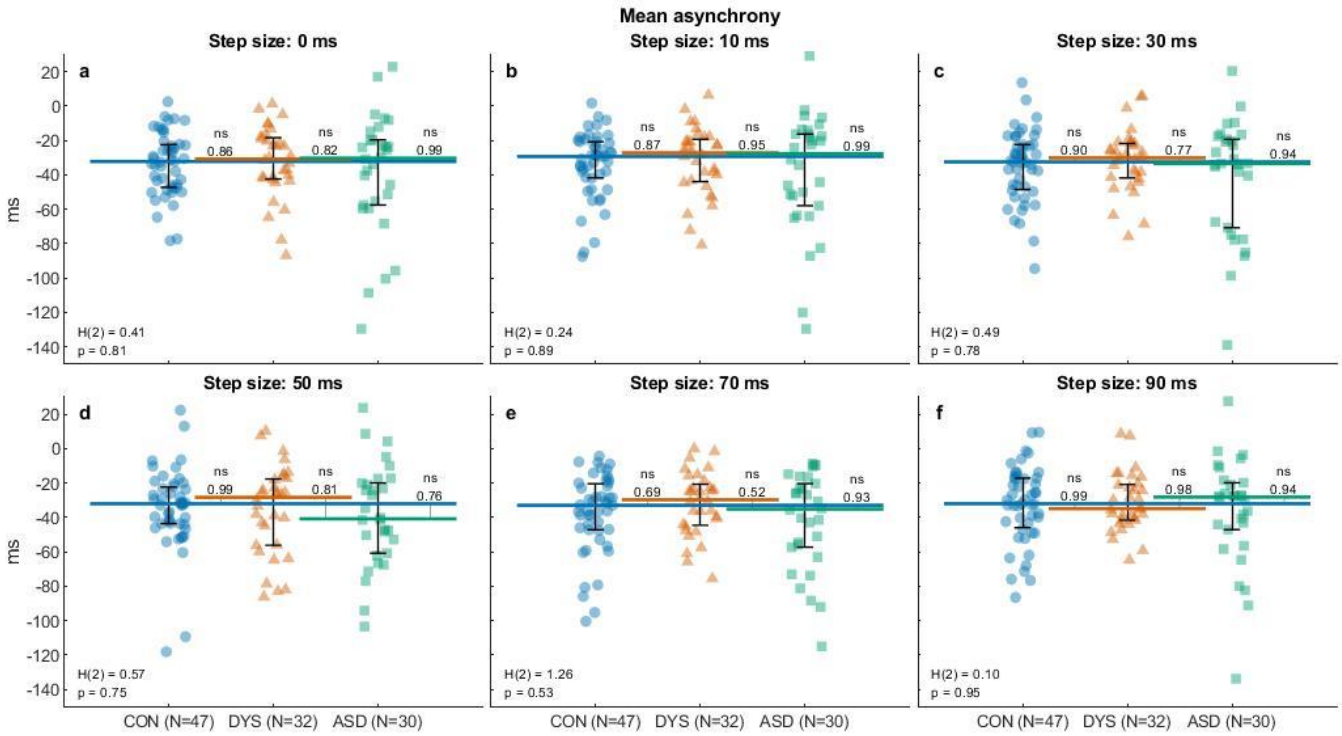

Supplementary Figure 3. No group differences were found in the negative mean asynchrony in both experiments. (a) Experiment 1: no tempo change (0 step-size). (b-f) Experiment 2: tempo changes, in step-sizes from 10ms to 90ms. No group differences emerge in any of the conditions. Each dot represents the standard deviation of one participant in a specific condition (average of the two repetitions). x-axis and color represent group membership (CON – control (neurotypical), DYS – dyslexia, ASD – autism; a small jitter was added to improve readability). The median of each group is denoted as a line of the same color; error bars around this median denote interquartile range. Kruskal-Wallis H-statistic and corresponding p-value are in the bottom-left corner; p-values of comparisons between groups are next to the line connecting the groups' medians. P-values in the panels were not corrected for multiple comparisons across conditions. Source data are provided as a Source Data file.

Supplementary Note 2. Dynamics of phase correction using autoregressive modelling

We used an autoregressive model to predict the current asynchrony, consider linear dependencies with several asynchronies back, not just the most recent asynchrony. We used stepwise regression to determine the number of previous asynchronies to use in predicting each asynchrony, both at the group level and at the single participant level. In the group model separate predictors were used for each participant, but the decision of whether to add a predictor or not was based on a group level criterion. The final model included three previous asynchronies, for all groups. At the single participant level, we found that for the majority of participants (95/109) the final model included one or two predictors, meaning that it was sufficient to use asynchronies one or two taps back to predict the current asynchrony, and no additional information was given by adding more asynchronies as predictors. For 11 of the remaining participants the final model included three or four predictors, and for 3 participants it included 5 predictors or more (we limited our model to 5 predictors a priori, Supplementary Figure 4a). There was no difference between the groups with regard to the number of predictors in the final model (χ^2(2,N=109)=8.22, p>0.4). This indicates that error correction in paced tapping to an isochronous metronome is a fast mechanism which depends only on very recent history. To further validate this point, we fit for all participants an autoregressive model with four predictors, asynchronies *k-1*, *k-2*, *k-3* and *k-4*, to predict the current asynchrony. We fit the model for each participant separately and compared the fitted coefficients across the groups (Supplementary Figure 4b). In accordance with the correlation results, we found a significant difference between the groups in the contribution of the most recent asynchrony to the current asynchrony (coefficient corresponding to the most recent trial (b1) - median [interquartile range]: neurotypical: 0.43 [0.26], dyslexia: 0.42 [0.28], autism: 0.57 [0.3]; H(2)=6.16, p=0.046), indicating that the most recent error is a better predictor of current error in the ASD group compared with each of the other groups, or in other words, the ASD group corrected less of the most recent error and carried a larger fraction to the next tap. Complementing the hierarchical regression results, the contribution of earlier asynchronies decayed quickly in all groups, with only a small contribution of asynchrony *k-2* to asynchrony *k* (*b2* median [interquartile range]: neurotypical: 0.06 [0.15], dyslexia: 0.07 [0.12], autism: 0.12 [0.18]), and even smaller for asynchrony *k-3* (*b3* median [interquartile range]: neurotypical: 0.02 [0.12], dyslexia: 0.06 [0.16], autism: -0.01 [0.15]). The contribution of asynchrony *k-4* did not significantly differ from zero in any group (*b4* median [interquartile range]: neurotypical: 0 [0.11], dyslexia: -0.02 [0.11], autism: -0.02 [0.13]; Wilcoxon signed rank test, p>0.2 for all 3 groups). No coefficients other than *b1* differed between the groups (all p>0.4, uncorrected). To summarize, error correction is a rapid mechanism, with asynchrony errors being corrected just a few seconds after they are committed. This fast correction is reduced in ASD, and intact in dyslexia, in line with previous observation of slow updates of perceptual observations in ASD, and not in dyslexia (Lieder et al., 2019).

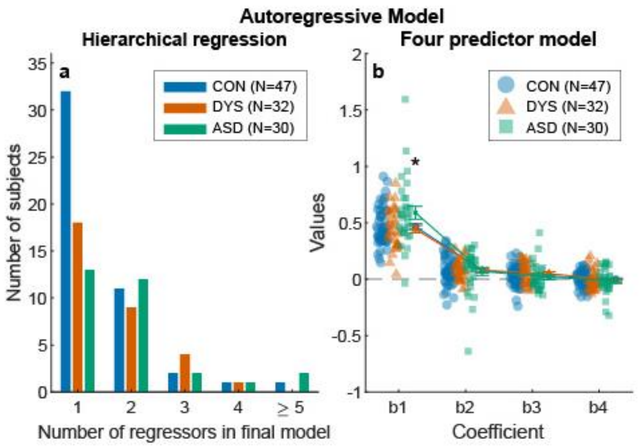

Supplementary Figure 4. Autoregressive model of phase correction shows that phase is a fast process, influenced only by recent errors. (a) Hierarchical regression fitted for each participant separately to predict the current phase error (asynchrony) using previous errors (each time going one tap earlier). For the majority of participants (106/109) no additional information to predict phase errors can be gained by using more than four preceding taps. CON – control (neurotypical), DYS – dyslexia, ASD – autism. (b) We modeled phase errors using the preceding four asynchronies for each participant separately. Each dot represents the coefficient value for one participant. Symbols connected by lines are the group means (error bars denote SEM). In agreement with the hierarchical regression results, this revealed that phase correction relies only on the most recent information (<2 seconds; for all three groups the last coefficient was not different from zero). Additionally, we found a significant difference between the groups in the contribution of the most recent asynchrony to the current asynchrony (b1; Kruskal-Wallis test, H(2)=6.16, p=0.046 (uncorrected)) in agreement with the consecutive correlation results (Figure 2). Source data are provided as a Source Data file.

Supplementary Figure 5. Responses to small tempo changes (10, 30ms)

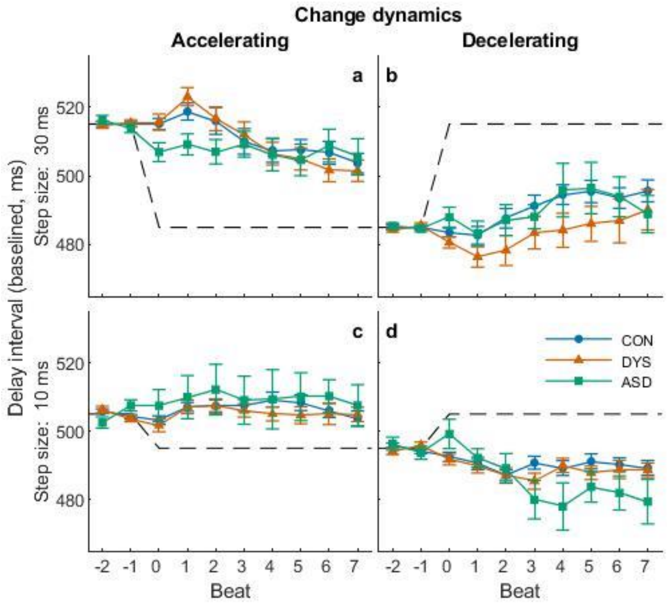

Supplementary Figure 5. Small tempo changes are unnoticed by participants (a-b) 30ms step-size and (c-d) 10ms step-size. x-axis represents the metronome-beat number around the moment of change (marked as 0), and y-axis measures the delay interval in each beat aligned to the pre-change metronome (mean group values, +/- SEM; values were calculated by first averaging responses within each participant and then across the group (CON – control (neurotypical), DYS – dyslexia, ASD – autism); error bars denote SEM across participants). Participants in all groups do not correct deviations in these step-sizes, because the changes are too small to initiate period correction mechanisms (Repp, 2001). Sample sizes: (a-b) 30ms step-size: N_{CON} =47, N_{DYS} =32, N_{ASD} =29. (c-d) 10ms step-size: N_{CON} =47, N_{DYS} =32, N_{ASD} =30. See Methods for the exclusion criteria. Source data are provided as a Source Data file.

Supplementary Note 3. Computational models parameter recovery

To ensure that our fitting procedure was capable of identifying individual differences in the computational modelling parameters, we performed parameter recovery analysis (following the procedure suggested in Van der Steen et al. 2015 and others). In both experiments, we used the fitted values from the experimental data (Figures 3 and 6) to simulate 1000 finger tapping blocks per subject and condition. For simulations of changing tempos, we used the same stimulus parameters which the subjects received in the task (the same starting tempo and timing of tempo changes). Before fitting the model, we projected the simulated data to the same procedures that were performed on the experimental data, namely, we excluded taps that were outside of a 400ms window around the simulated metronome beat and excluded blocks where more than 40% of the taps were removed. The isochronous simulated blocks (Experiment 1) were fitted with a model without period correction. For the simulated tempo changes blocks (Experiment 2), we extracted only the taps surrounding a tempo change (from two taps before the change, to seven taps following the change, as in the experimental data) and fitted each separately.

For both models, the recovered values were extremely close to the parameter values used for the simulation. For the isochronous blocks (Experiment 1, no period correction): Spearman correlations between the recovered and the fitted data for the phase correction parameter (α) were $\rho_{CON} = 0.95$, $\rho_{DYS} = 0.92$ and $\rho_{ASD} = 0.98$ in the separate groups, and $\rho = 0.96$ when considering all participants together (Supplementary Figure 6). For the timekeeper noise parameter correlations were all above $\rho = 0.94$ in the separate groups, and $\rho = 0.96$ when considering all participants together, and for the motor noise parameter correlations all above $\rho = 0.93$ in the separate groups, and $\rho = 0.94$ when considering all participants together (all correlations were highly significant, $p < 0.0001$). RMSE (root mean square error) values were below 0.07 arb. units for the phase correction parameter (α) in all three groups, below 2ms for the timekeeper parameter in all three groups and below 3ms for the motor noise parameter in all three groups.

For the alternating blocks (Experiment 2), the Spearman correlations between recovered and fitted data for the phase correction parameter (α) were above $\rho = 0.94$ for each of the three groups, and when considering all participants together in all three step-sizes. For the period correction parameter (β) Spearman correlations between recovered and fitted data in the were above $\rho = 0.98$ for each of the three groups, and when considering all participants together in all three step-sizes (Figure S6). RMSE values for both error correction parameters were also very low – under 0.089 for phase correction and 0.063 for the period correction parameter in all groups and step-sizes. The timekeeper noise parameter also showed good parameter recovery – Spearman correlations were above 0.91 and RMSE was below 8.6ms in all groups and step-sizes. The Spearman correlations of the motor noise recovered values with the fitted values were lower, between $\rho = 0.55$ and $\rho = 0.83$, but RMSE values were generally low (all under 5.1ms). The combined measures (calculated by z-scoring, as for the fitted experimental data) showed high correlations as well. For the phase correction parameter and timekeeper noise parameter Spearman correlations were larger than $\rho = 0.95$ across groups and when combining all together, for the period correction

parameter Spearman correlations were larger than $\rho = 0.97$ across groups and when combining all together. For the motor noise parameter correlations were lower, but all above $\rho = 0.75$ (all correlations, including the motor noise correlations, were highly significant, $p < 0.0001$). The recovered combined update rate (calculated by z-scoring the recovered phase correction parameter from Experiment 1 (α) and recovered period correction parameter from experiment 2 (β)) was highly correlated with the fitted value, with correlations between $\rho = 0.94$ and $\rho = 0.98$ in the separate groups and when combining all participants together (Supplementary Figure 6).

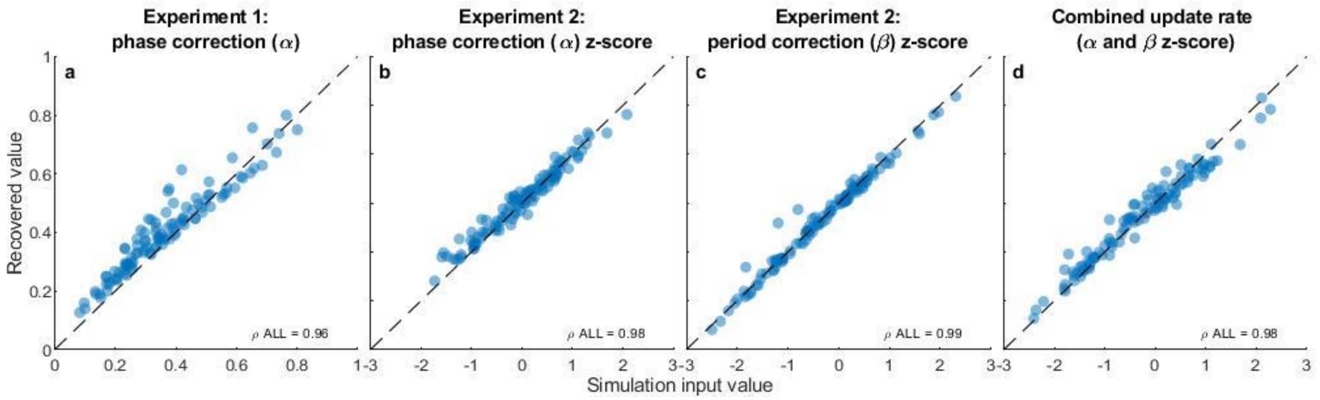

Supplementary Figure 6. Parameter recovery of the error correction parameters from both experiments. (a) Experiment 1 phase correction (α), (b) Experiment 2 phase correction (z-score combined value across blocks), (c) Experiment 2 period correction (z-score combined value across blocks) and (d) Combined update rate (averaging phase and period correction). All error correction parameters showed remarkable similarity between the values used for the simulation (which were the fitted values of each subject), and the recovered values. Each dot represents the average value of one simulation setting (corresponding to one subject), calculated using 1000 simulations. The dashed line represents equality between the recovered and the fitted values. All points are remarkably close to the equality line. Error correction parameters in specific conditions, and the timekeeper parameter all showed similar patterns. Motor noise recovery was noisier in Experiment 2, but nonetheless very close to the values used to generate the data (see values in the text). Source data are provided as a Source Data file.

Supplementary Note 4. The model reproduces tapping profiles of the different groups

For each subject, we simulated 500 repetitions of each experiment based on the fitted parameter values for both computational models. As in the experiment, each condition was repeated twice, resulting in 1000 simulated blocks of each condition. In Experiment 1 (Supplementary Figure 7) we calculated the correlation between consecutive simulated asynchronies, which depict the amount of error correction performed by participants (higher values indicated errors are carried for a longer time). The simulated values were highly matched to the observed experimental values (Supplementary Figure 7: across groups Spearman correlation is $\rho = 0.94$, single group correlations are all larger than 0.9). We find a significant difference between the simulated groups' data (Kruskal-Wallis test, p=0.043, Supplementary Figure 7b), though the difference between neurotypical and autism and between dyslexia and autism was only marginal (p=0.064 and p=0.071 respectively). The effect size observed in the experiment (H(2) = 8.25, Supplementary Figure 7c) was not significantly different from the distribution of effect sizes across our simulated experiments (p=0.11). In Experiment 2, we extracted the segments around changes in tempo, as was done for Figure 4 in the main text. The simulated data (Supplementary Figure 8) reproduces the patterns observed in the experiment. Specifically, simulations based on data fitted from neurotypical individuals and individuals with dyslexia show quick detection and correction for changes in tempo (especially for the prominent changes in the 70ms and 90ms step-sizes). In addition, simulations based on the data of individuals with autism show only partial updating to the change and continues to show a marked difference in asynchrony even 7 taps after the tempo change. As in the experiment data, the dyslexia simulated values for the 50ms step-size are midway between the autism and neurotypical values.

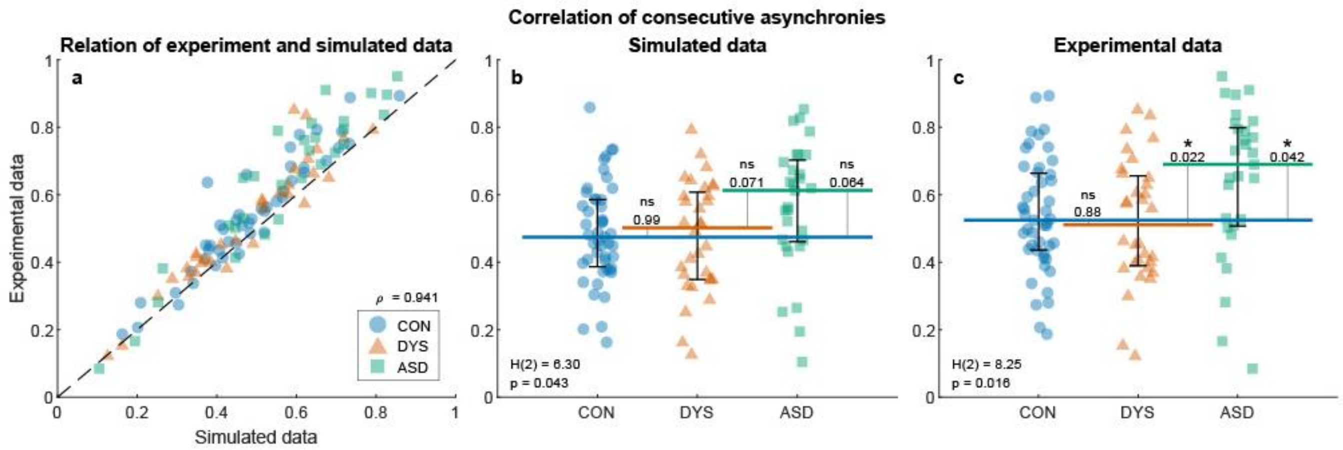

Supplementary Figure 7. The model reproduces the error correction patterns observed in Experiment 1. Each dot represents values corresponding to one participant (CON – control (neurotypical), DYS – dyslexia, ASD – autism; N=108 subjects (N_{CON}=47, N_{DYS}=32, N_{ASD} =29), one ASD participant was excluded due to insufficient taps; simulated value was averaged over 500 simulation repetitions). (a) Simulated correlations of consecutive asynchronies are very close to the correlations observed in the experiment. The dashed line represents equality between the simulated and observed values in the experiment. All points are remarkably close to the equality line. Spearman correlation between the observed and simulated values is in the bottom right corner. (b)

Comparison of simulated asynchrony correlations between groups reveals a significant group difference, although post-hoc comparison between pairs of groups were marginal. (c) Comparison of observed asynchrony correlations between groups. Note that this is slightly different than the values in Figure 2d, since subjects for whom the model could not be fitted are not present in this plot (a total of 3 blocks, see Methods). In panels (b) and (c) x-axis and color represent group membership (a small jitter was added to improve readability). The median of each group is denoted as a line of the same color; error bars around this median denote interquartile range. KruskalWallis H-statistic and corresponding p-value are in the bottom-left corner; p-values of comparisons between groups are next to the line connecting the groups' medians. Source data are provided as a Source Data file.

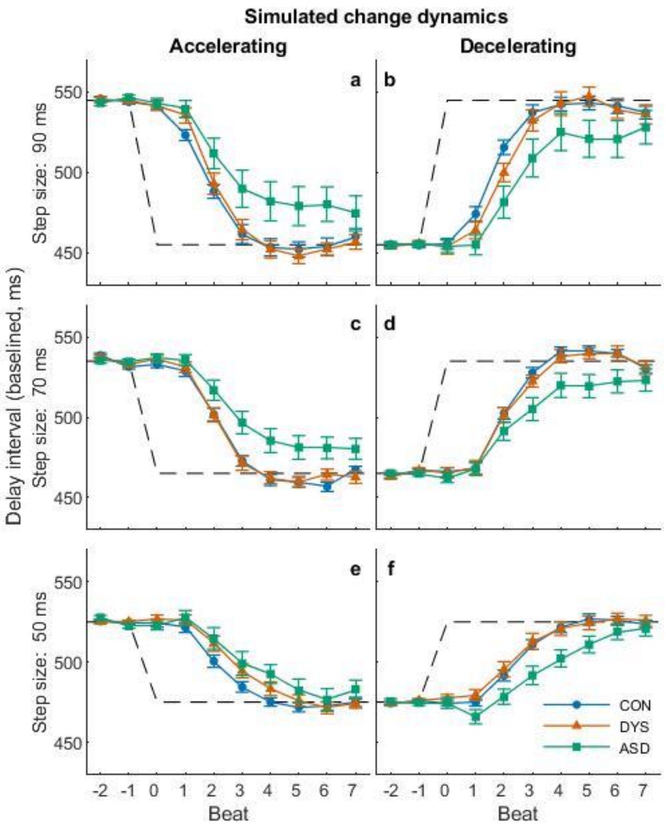

Supplementary Figure 8. Simulation of the model fitted to the data (compare with Figure 4). Simulated changes in tempo reproduce the patterns observed in the Experiment, namely, individuals with autism adapt to changes in tempo only partially, even when changes are very salient**.** (a-b) 90ms step-size, (c-d) 70ms step-size and (e-f) 50ms step-size. In each panel the x-axis represents the metronome-beat number around the moment of tempo change (beat 0), and the y-axis measures the simulated delay interval in each beat (mean across participants, error bars denote SEM across simulations of different participants). As in the experimental data, large changes (70, 90ms) are quickly corrected by the simulations of the neurotypical (CON, control) and dyslexia (DYS) groups, but not by simulated data based on the autism (ASD) fitted values. This difference is less pronounced for the 50ms step-size, as in the experimental data. N=109 subjects (N_{CON} =47, N_{DYS} =32, N_{ASD} =30), except one participant with dyslexia in the 90ms step-size and one neurotypical participant in the 50ms step-size who did not have a sufficient number of taps to be included in the computational modelling. Source data are provided as a Source Data file.

Supplementary Table 1. Cognitive profile. All participants (neurotypical N=47, dyslexia N=32, ASD N=30) completed a set of cognitive assessments evaluating general reasoning skills (standard Block Design and Digit Span tasks, WAIS-IV) and reading abilities. Values are median [interquartile range]. The groups are matched on age and reasoning skills. Digit span score is lower in dyslexia compared with the other two groups (in agreement with previous results, e.g. Helland & Asbj∅rnsen, 2004). Reading accuracy on single words and pseudowords is significantly poorer in the dyslexia group compared with the other two groups, while the rate is slower in both dyslexia and autism groups. Finally, accuracy in paragraph reading is comparable between autism and dyslexia groups, likely due to the beneficial influence of context in this kind of tasks, which we show to be impaired in autism. Source data are provided as a Source Data file.

Test			Neurotypical	Dyslexia	Autism	Kruskal-Wallis test H statistic (p value)	Tukey-Kramer poster-hoc H0 rejected
General	Age (years)		25 [3]	24 [3.5]	25.5 [9]	1.28 (0.5)	
	Block Design (scaled score)		12 [3.8]	12 [3]	10 [4]	5.64 (0.06)	
	Digit Span (scaled score)		10 [3.8]	7 [2]	8.5 [3]	26.45 ($<10^{-5}$)	Neurotypical-Dyslexia<br>Dyslexia-Autism
Reading	Words	Accuracy (% correct)	100 [4.2]	87.5 [8.3]	95.8 [8.3]	25.58 ($<10^{-5}$)	Neurotypical-Dyslexia<br>Dyslexia-Autism
		Rate (words/min)	102.8 [38.5]	72.5 [31.4]	71.5 [36.4]	22.56 ($<10^{-4}$)	Neurotypical-Dyslexia<br>Neurotypical-Autism
	Pseudowords	Accuracy (% correct)	91.7 [15.6]	58.3 [33.3]	87.5 [25]	34.41 ($<10^{-7}$)	Neurotypical-Dyslexia<br>Dyslexia-Autism
		Rate (words/min)	60.4 [25.5]	35.5 [17.2]	39.7 [21.3]	28.12 ($<10^{-6}$)	Neurotypical-Dyslexia<br>Neurotypical-Autism
	Paragraph	Accuracy (% correct)	98.7 [3.1]	95.1 [3.9]	96.4 [5.2]	21.05 ($<10^{-4}$)	Neurotypical-Dyslexia<br>Neurotypical-Autism
		Rate (words/min)	134.9 [21.7]	102.9 [18.3]	101.5 [46.6]	37.32 ($<10^{-8}$)	Neurotypical-Dyslexia<br>Neurotypical-Autism

Supplementary Note 5. bGLS estimation algorithm for data with missing values

The original estimation algorithm from Jacoby et al. (2015) is not applicable when some taps are missing. We adapted the model to enable fitting with missing values, by adding an additional step (no. 6) after the calculation of the covariance matrix to remove the rows and columns corresponding to the indices of missing values so that only the relevant dependencies are inserted into the model. The method is otherwise identical to that provided in Jacoby et al. (2015). The following procedure uses the notations from Jacoby et al., namely $\mathbf{y}$ = the vector of inter-tap-intervals (demeaned), and $\mathbf{B}$ = the vector of asynchronies (also demeaned). We denote the missing values indices by $\mathbf{m}$. Remove the missing values from $\mathbf{y}$ and $\mathbf{B}$. N_1 = length of $\mathbf{y}$ before removing missing values; N_2 = length after removing missing values. Initialize by setting the covariance matrix to the identity matrix ($\Sigma_1 = I_{N_2 * N_2}$).

For each iteration n:

1) Compute general least squares: $x_n = (\mathbf{B}^T \Sigma_n^{-1} \mathbf{B})^{-1} (\mathbf{B}^T \Sigma_n^{-1}) \mathbf{y}$
2) Set: $\mathbf{c}_n = \mathbf{y} - \mathbf{B}\mathbf{x}_n$
3) Calculate: $\gamma_n(0) = \frac{1}{N_2} \sum_{k=1,\ldots,N_2} \mathbf{c}_n(k)^2$ and $\gamma_n(1) = \frac{1}{N_2 - 1} \sum_{k=1,\ldots,N_2-1} \mathbf{c}_n(k) \mathbf{c}_n(k+1)$
4) Adjust them so the bound applies: $0 < -\gamma_n(1) < \gamma_n(0) + 2\gamma_n(1)$. This step is needed to ensure reliable estimation, see Jacoby et al., 2015.
5) Calculate the new covariance matrix: $\Sigma_{n+1} = \gamma_n(0) I + \gamma_n(1) \Delta$, when I is the N_1 by N_1 identity matrix and Δ is an N_1 by N_1 matrix with one on the two secondary diagonals and 0 elsewhere.
6) Adjust the covariance by removing the columns and rows of the indices of the original missing values (using $\mathbf{m}$), so the resulting matrix is now size N_2 by N_2.

After the last iteration extract the estimated parameters according to:

1) $\alpha = -x_n$
2) $\sigma_{\mathrm{T}} = \sqrt{\gamma_n(0) + 2\gamma_n(1)}$
3) $\sigma_{\mathrm{M}} = \sqrt{-\gamma_n(1)}$

Note that if $-\gamma_n(1)$ is negative we set $\sigma_{\mathrm{M}} = 0$. This produce small bias for small values of σ_{M} as the noisy estimate can be negative. However, the absolute estimation error is still small (see Supplementary Note 3 and Jacoby et al. 2015).

A similar procedure can be done to fit the extended model with missing values, but since we used only short segments around the time of changes in tempo, we did not include segments with missing values in the estimation procedure.

References

1. Lieder, I., Adam, V., Frenkel, O., Jaffe-Dax, S., Sahani, M. & Ahissar, M. Perceptual bias reveals slow-updating in autism and fast-forgetting in dyslexia. *Nature Neuroscience* **22**, 256-264 (2019).
2. Repp, B. H. Processes underlying adaptation to tempo changes in sensorimotor synchronization. *Human movement science* **20**, 277-312 (2001).
3. Van Der Steen, M.M., Jacoby, N., Fairhurst, M.T. & Keller, P.E. Sensorimotor synchronization with tempo-changing auditory sequences: Modeling temporal adaptation and anticipation. *Brain research* **1626**, 66-87 (2015).
4. Helland, T. & Asbj∅rnsen, A. Digit span in dyslexia: Variations according to language comprehension and mathematics skills. *Journal of Clinical and Experimental Neuropsychology* **26**, 31-42 (2004).
5. Jacoby, N., Tishby, N., Repp, B. H., Ahissar, M. & Keller, P. E. Parameter estimation of linear sensorimotor synchronization models: phase correction, period correction, and ensemble synchronization. *Timing & Time Perception* **3**, 52-87 (2015).
