# Supplementary material for: Slow update of internal representations impedes synchronization in autism
Source: Nat Commun. 2021 Sep 14;12:5439. doi: 10.1038/s41467-021-25740-y (PMC8440645; doi:10.1038/s41467-021-25740-y)
Supplement: Supplementary file 3 — Reporting Summary [file 41467_2021_25740_MOESM3_ESM.pdf]

nature portfolio

Corresponding author(s): Merav Ahissar

Last updated by author(s): 18/06/2021

Reporting Summary

Nature Portfolio wishes to improve the reproducibility of the work that we publish. This form provides structure for consistency and transparency in reporting. For further information on Nature Portfolio policies, see our Editorial Policies and the Editorial Policy Checklist.

Statistics

For all statistical analyses, confirm that the following items are present in the figure legend, table legend, main text, or Methods section.

n/a	Confirmed	
☐	☒	The exact sample size (n) for each experimental group/condition, given as a discrete number and unit of measurement
☐	☒	A statement on whether measurements were taken from distinct samples or whether the same sample was measured repeatedly
☐	☒	The statistical test(s) used AND whether they are one- or two-sided<br>*Only common tests should be described solely by name; describe more complex techniques in the Methods section.*
☐	☒	A description of all covariates tested
☐	☒	A description of any assumptions or corrections, such as tests of normality and adjustment for multiple comparisons
☐	☒	A full description of the statistical parameters including central tendency (e.g. means) or other basic estimates (e.g. regression coefficient) AND variation (e.g. standard deviation) or associated estimates of uncertainty (e.g. confidence intervals)
☐	☒	For null hypothesis testing, the test statistic (e.g. F, t, r) with confidence intervals, effect sizes, degrees of freedom and P value noted<br>*Give P values as exact values whenever suitable.*
☒	☐	For Bayesian analysis, information on the choice of priors and Markov chain Monte Carlo settings
☐	☒	For hierarchical and complex designs, identification of the appropriate level for tests and full reporting of outcomes
☐	☒	Estimates of effect sizes (e.g. Cohen's d, Pearson's r), indicating how they were calculated

Our web collection on statistics for biologists contains articles on many of the points above.

Software and code

Policy information about availability of computer code

Data collection	Data was collected using the open-source software audacity: https://www.audacityteam.org/
Data analysis	The custom code used to analyze the data in this study (including the implementation used for the bGLS algorithm) and create all figures (except Figures 1a, 3a and 6a) is available publicly using the following repository: 10.5281/zenodo.4930034. Matlab version: 2019b.

For manuscripts utilizing custom algorithms or software that are central to the research but not yet described in published literature, software must be made available to editors and reviewers. We strongly encourage code deposition in a community repository (e.g. GitHub). See the Nature Portfolio guidelines for submitting code & software for further information.

Data

Policy information about availability of data

All manuscripts must include a data availability statement. This statement should provide the following information, where applicable:
- Accession codes, unique identifiers, or web links for publicly available datasets
- A description of any restrictions on data availability
- For clinical datasets or third party data, please ensure that the statement adheres to our policy

All data generated in this study have been deposited in the OSF public repository under accession code: osf.io/83wnu. Source data are provided with this paper.

Field-specific reporting

Please select the one below that is the best fit for your research. If you are not sure, read the appropriate sections before making your selection.

☐ Life sciences ☒ Behavioural & social sciences ☐ Ecological, evolutionary & environmental sciences

For a reference copy of the document with all sections, see nature.com/documents/nr-reporting-summary-flat.pdf

Behavioural & social sciences study design

All studies must disclose on these points even when the disclosure is negative.

Study description	Computational modeling of new behavioral data collected in 3 populations (neurotypical, dyslexia, autism).
Research sample	Data were collected from 133 participants (56 neurotypical, 39 dyslexia, 38 autism). The final group consisted of 109 participants (47 neurotypical, 30 female; 32 dyslexia, 19 female; 30 autism, 5 female). Age (median [interquantile range]): neurotypical - 25 [3], dyslexia - 24 [3.5], autism 25.5 [9]. The size of the groups is larger than many other studies in the field, and was intended to capture a representative sample of all populations. Additionally, participants with autism were recruited from multiple sources to ensure the sample is representative.
Sampling strategy	Participants in all groups were randomly sampled. We recruited participants to all groups within a predefined time period, and it was decided to continue recruitment only if one of the groups contained less than 20 participants (customary in the field). By the end of the recruitment period all groups were larger than 20 participants. Neurotypical participants and participants with dyslexia were recruited through advertisements at the Hebrew University of Jerusalem and colleges near the university. Participants with ASD were recruited through clinics, designated facilities, and support groups. All participants were native Hebrew speakers. We recruited only participants with minimal musical experience (less than 2 years of self-reported musical education). All participants in the dyslexia group had been diagnosed by authorized clinicians as having a specific reading disability and all participants with ASD were diagnosed with autism spectrum disorders (including autism, Asperger, and PDD-NOS) by expert clinicians.
Data collection	Tapping was performed on a custom-made wooden box, including a microphone which recorded the participant's responses. We used either Focusrite Saffire 6 USB or Focusrite Scarlett 2i2 sound cards, which simultaneously recorded the output from the microphone installed inside the box and a split of the headphone signal, so that the playback latency and jitter could be estimated for each recording. Researchers were present during the demo block, but usually left the room for the experimental session, except rare cases when the testing conditions did not enable this. The researchers were not blind to the hypothesis or condition during collection.
Timing	Piloting began in November 2013. The collection of data from neurotypical and dyslexia participants took place between March 2014 and September 2015, and between May 2017 and March 2018. ASD data collection took place between December 2015 and March 2018.
Data exclusions	Prior to data collection, it was decided to match the groups on reasoning skills, while excluding the minimal number of individuals with autism (which were most difficult to recruit). The specific exclusion policy was based on the participants we managed to recruit within the allocated time. We excluded all participants with a Block Design score (Weschler, 2008) below 7 (1 dyslexia, 6 autism), and neurotypical and dyslexia participants with Block Design above 15 (7 neurotypical, 4 dyslexia).<br>As an additional pre-established measure to verify reading skills pertaining to the recruitment of the dyslexia group we assessed reading-related measures in the lab, leading us to exclude one neurotypical participant and two participants with dyslexia.<br>Finally, 3 participants were excluded due to extreme mean asynchrony values (>50ms, 1 neurotypical, 2 autism), suggesting a very different tapping strategy than that of other participants. This is based on previous finger tapping studies.
Non-participation	None
Randomization	Participants with no diagnosed psychiatric or neurological disorders were allocated to the neurotypical group. Participants with a specific reading disability were allocated to the dyslexia group. Participants diagnosed with autism spectrum disorders (autism, Asperger or PDD-NOS) were allocated to the autism group.

Reporting for specific materials, systems and methods

We require information from authors about some types of materials, experimental systems and methods used in many studies. Here, indicate whether each material, system or method listed is relevant to your study. If you are not sure if a list item applies to your research, read the appropriate section before selecting a response.

Materials & experimental systems

n/a	Involved in the study	
☒	☐	Antibodies
☒	☐	Eukaryotic cell lines
☒	☐	Palaeontology and archaeology
☒	☐	Animals and other organisms
☐	☒	Human research participants
☒	☐	Clinical data
☒	☐	Dual use research of concern

Methods

n/a	Involved in the study	
☒	☐	ChIP-seq
☒	☐	Flow cytometry
☒	☐	MRI-based neuroimaging

Human research participants

Policy information about studies involving human research participants

Population characteristics	See above.
Recruitment	Neurotypical participants and participants with dyslexia were recruited through advertisements at the Hebrew University of Jerusalem and colleges near the university. Participants with ASD were recruited through clinics (including author T.E.), designated facilities, and support groups. Multiple recruitment sources were used to balance any potential biases that each single source might have. All participants were native Hebrew speakers. We recruited only participants with minimal musical experience (less than 2 years of self-reported musical education). All participants in the dyslexia group had been diagnosed by authorized clinicians as having a specific reading disability and all participants with ASD were diagnosed with autism spectrum disorders (including autism, Asperger, and PDD-NOS) by expert clinicians.
Ethics oversight	All experiments were approved by the Ethics Committee of the Psychology Department of the Hebrew University and the Helsinki Ethics Committee of Sheba Hospital (required for testing individuals with ASD recruited through their adult clinic).

Note that full information on the approval of the study protocol must also be provided in the manuscript.
